# Systematic review of health state utility values for economic evaluation of colorectal cancer

**DOI:** 10.1186/s13561-016-0115-5

**Published:** 2016-08-19

**Authors:** Kim Jeong, John Cairns

**Affiliations:** Department of Health Services Research and Policy, London School of Hygiene and Tropical Medicine, 15-17 Tavistock Place, London, WC1H 9SH UK

**Keywords:** Health state utility value, Colorectal cancer, QALY, Economic evaluation

## Abstract

Cost-utility analyses undertaken to inform decision making regarding colorectal cancer (CRC) require a set of health state utility values (HSUVs) so that the time CRC patients spend in different health states can be aggregated into quality-adjusted life-years (QALY). This study reviews CRC-related HSUVs that could be used in economic evaluation and assesses their advantages and disadvantages with respect to valuation methods used and CRC clinical pathways. Fifty-seven potentially relevant studies were identified which collectively report 321 CRC-related HSUVs. HSUVs (even for similar health states) vary markedly and this adds to the uncertainty regarding estimates of cost-effectiveness. There are relatively few methodologically robust HSUVs that can be directly used in economic evaluations concerned with CRC. There is considerable scope to develop new HSUVs which improve on those currently available either by expanded collection of generic measures or by making greater use of condition-specific data, for example, using mapping algorithms.

**Electronic supplementary material:**

The online version of this article (doi:10.1186/s13561-016-0115-5) contains supplementary material, which is available to authorized users.

## Introduction

Colorectal cancer (CRC) is the third most commonly diagnosed cancer worldwide [1]. CRC was traditionally more common in the western world but some Asian countries have shown an increase in CRC incidence in recent years [2]. Economic evaluation to inform decision making regarding CRC requires a set of health state utility values (HSUVs) so that the time CRC patients spend in different health states can be aggregated into quality-adjusted life-years (QALYs).

There are four ways by which the required HSUVs can be empirically generated:

1. There are generic preference-based measures (PBM), such as the EQ-5D, SF-6D, 15D and the HUI3, where generic health states are valued using a tariff based on the preferences of the general public elicited using methods such as the time trade-off (TTO) and the standard gamble (SG).
2. An alternative approach is to identify a number of relevant cancer-specific health states (as opposed to using generic health state descriptions) and to value these health states directly, again using methods such as the TTO and the SG. In this case the valuations are potentially made by cancer patients themselves, health care professionals or the general public.
3. A variation on this second approach is to develop a preference-based algorithm with which a full range of cancer-specific health states can be valued. Two such measures, the EORTC-8D and the QLU-C10D, are based on items from the Quality of Life Questionnaire C30 (QLQ-C30).
4. Finally, a mapping algorithm can be used to transform cancer-specific data such as the EORTC QLQ-C30 and the Functional Assessment of Cancer Therapy-General (FACT-G) into generic PBMs.

This paper reviews CRC-related HSUVs that could be used in economic evaluations and assesses their advantages and disadvantages with reference to the valuation methods used and CRC clinical pathways.

## Review

### Methods

The literature was searched to identify CRC-related HSUVs for use in economic evaluation. MEDLINE, MEDLINE In-Process & Other Non-Indexed Citations, Embase (up to 30 October 2015) and Health Economic Evaluations Database (HEED, up to December 2014) were searched using the keywords colorectal cancer, health-related quality of life, QALY and economic evaluation. The search was restricted to studies in English. The search was broadened to include the National Institute for Health and Care Excellence (NICE) website (www.nice.org.uk) to minimise the chance of missing relevant studies. Economic filters were used when searching for evidence on generalist databases, such as MEDLINE. A simplified search was undertaken without using economic filters, for evidence on economics databases such as HEED. A further search was run on non-economic databases, including MEDLINE, to capture studies that are relevant to mapping. Search strategies are reported in Appendix 1. Relevant conference abstracts were tracked for full journal publications. All search results were downloaded into EndNote and duplicates removed. Titles and abstracts were screened between two independent reviewers and full papers that did not meet the inclusion criteria were excluded. The study selection criteria are reported in Appendix 2. Studies were included if they contained CRC-related HSUVs which had not been previously reported, be they generic PBMs or directly valued CRC-related health state descriptions, or mapping to generic PBMs based on direct statistical association mapping.

Full text was acquired for the remaining studies (including those which had insufficient details, such as no abstract). All included studies were read and any disagreements were resolved by discussion between the two reviewers. Of the 285 papers identified as potentially relevant 228 were excluded because they did not report CRC-related HSUVs but presented psychometric validation studies without internal validation properties, the values were previously reported in other included studies, they involved unspecified or not clearly specified CRC-related health state utility values, or a primary mapping function was not reported.

**Fig. 1:**
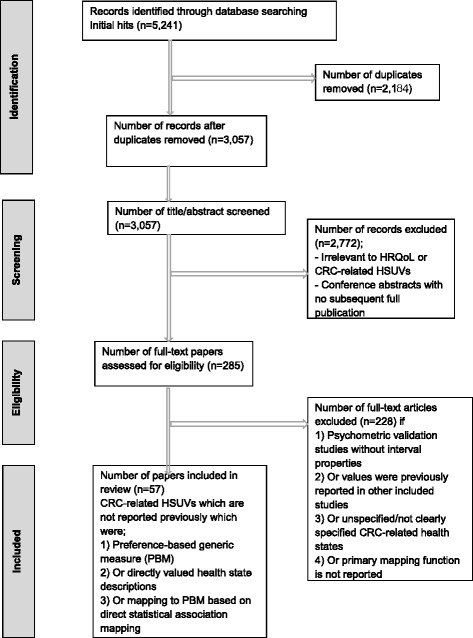
Numerical summary of the searches for the review

A total of 57 studies were included in the reviews (see Additional file 1). The numerical summary of the search and selection process for the review is reported in Fig. 1.

Descriptive characteristics (year of publication, country of origin, intervention type, number and mean age of respondents) and methodological characteristics (what was the measure of value; how was the health state described and valued; who valued it; how the QALY was aggregated) were collected for the 57 studies [3]. Findings from selected studies are discussed in the following section.

### Results

Of the 57 studies, eleven were set in the US [4–14], eight in the UK [15–22], seven in the Netherlands [23–29], five in Hong Kong [30–34], four in Canada [35–38], two each in Norway, Korea, Australia and Japan [39–46] and other country settings included Spain, Germany, India, Malaysia, Singapore, Sweden and Turkey [47–54]. Some studies did not report their settings or were multinational or multicentre studies [55–60]. Studies were mostly published in the last 15–20 years and focussed narrowly on different interventions at specific stages of CRC. For example, the adverse events (AEs) of chemotherapy and survival (partial response) in metastatic CRC (mCRC) were the main condition of interest in several studies [14, 20, 36, 38, 44, 48–50, 56, 58, 59]. HSUVs associated with rectal cancer have been reported [7, 18, 23–25, 27, 52, 54].

The 57 studies included in this review reported a total of 368 CRC-related HSUVs. All reported HSUVs are summarised in Additional file 2.

#### Generic preference-based measures

Thirty-two studies collected health state information from CRC patients using generic PBMs, and have applied health state tariffs based on the preferences of the general public. These studies generally collect data from patients recruited to trials, usually several hundred patients and at multiple time points. The most widely reported generic PBM is the EQ-5D valued using the UK (TTO-derived) value set with some exceptions [28, 57, 58] followed by the SF-6D [6, 30, 33, 34, 37] and HUI3 [10, 11, 38].

#### Direct valuation of CRC health states

Fourteen studies directly valued CRC health states. Preferences have been elicited either using the TTO method with patients or a surrogate group [4, 5, 36, 44, 45, 48–50] or SG [7, 8, 13, 20, 35]. Generally, these studies have involved fewer than 100 respondents. The participants have been drawn from CRC patients, health care professionals and the community or general population (non-patient, non-health care professional). Only one study recruited a sample entirely from the general population [44] and one entirely from CRC patients [8]. Mean utility values from health care professionals were lower than those from patients across health states [8, 12]. The remission health state was valued similarly by both groups, whereas the community group assigned lower values to adjuvant therapy-related AEs [4].

#### Preference-based condition-specific measures

Another approach has recently been developed which offers an alternative to using directly valued health states from the literature. The EORTC-8D is a cancer-specific PBM derived from the EORTC QLQ-C30 [61]. It utilises ten items from the thirty items of the QLQ-C30. A total of 85 EORTC-8D health states were valued by 350 members of the UK general public and these responses were then modelled to let any of the EORTC-8D states be valued. The QLU-C10D utilises twelve QLQ-C30 items to produce a ten dimensional measure [62]. However, to date this approach has not been used to value CRC health states.

#### Mapping

The absence of data on generic PBMs from most cancer trials has generated considerable interest in mapping algorithms, from cancer-specific measures such as the Functional Assessment of Cancer Therapy-General (FACT-G) and the EORTC QLQ-C30 to measures such as the EQ-5D and the SF-6D [63, 64]. While several studies have reported mapping algorithms in the cancer area [9, 30, 32, 37, 41, 42, 47], only one of the mapping algorithms was developed using responses from CRC patients [65] and only one study has reported HSUVs for different CRC-related health states based on an algorithm [31]. The mapping studies are summarised in Additional file 3.

#### HSUVs and the clinical pathway

For the purposes of estimating QALYs it is usually necessary to have information on HSUVs at several points along the clinical pathway. Evaluations of screening or diagnosis require valuations at the time of the intervention and subsequently following treatment.

##### CRC Screening-related HSUVs

Only one study has reported CRC-screening related HSUVs as presented in Table 1 [26].

**Table 1:** CRC screening- related HSUVs

Valuation methods used	HSUVs reported	Reference
EQ-5D	Negative FS after positive FIT 0.81 Positive FS after positive FIT 0.82	Kapidzic [26]

*COL* colonoscopy, *CRC* colorectal cancer, *FIT* faecal immunochemical test, *FS* flexible sigmoidoscopy, *HSUVs* health state utility values

##### Colostomy-related HSUVs

Sixteen colostomy-related HSUVs were reported from 4 studies [5, 8, 12, 35]. Disutility of 0.09 [8] and of 0.111 [12, 35] were reported among rectal cancer patients with colostomy compared with those without colostomy respectively. The utility of having a stoma among former CRC patients with a reversed colostomy was 0.20 lower compared with those currently have a stoma [12]. HSUVs related to having a colostomy (surgery) and no colostomy (radiotherapy) were measured using SG in the primary treatment for rectal cancer. People with a colostomy assigned a higher value than people without a colostomy [35]. The summary of colostomy-related HSUVs is presented in Table 2.

**Table 2:** Colostomy-related HSUVs

Valuation methods used	HSUVs reported	Reference
SG	With colostomy 0.915 Without colostomy 0.804	Boyd^a^ [35]
TTO	[20 years with CRC; 20 years with a colostomy] Unscreened [0.80; 0.80] Screened [0.80; 0.75] Enrolled in a COL screening program [0.85; 0.79] CRC patients [0.83; 0.90]	Dominitz [5]
EQ-5D	With stoma 0.836 Without stoma 0.870	Hamashima [40]
SF-6D	With stoma 0.69 Without stoma 0.73	Hornbrook [6]
SG	Stage II/III rectal cancer, permanent colostomy 0.50 Stage IV metastatic/unresectable disease without colostomy 0.25 State IV metastatic/unresectable disease with colostomy 0.25	Ness [8]
TTO	Currently with colostomy 0.84 Reversed colostomy 0.64 Community members 0.63	Smith [12]
EQ-5D	PRT and TME PS 0.823 TME PS 0.853	van den Brink [27]
SG	Stage II/III RC treated with resection, chemotherapy, radiation therapy and with permanent ostomy 0.50	Ness [8]

^a^ Reported HSUVs are re-expressed on a 0–1 scale

*COL* colonoscopy, *CRC* colorectal cancer, *HSUVs* health state utility values; *RC* rectal cancer, *SG* standard gamble, *TTO* time trade-off, *PRT* preoperative radiotherapy, *TME* total mesorectal excision, *PS* permanent stoma

##### HSUVs and colorectal polyps

Wong and colleagues [30] reported two HSUVs using SF-6D for those individuals with low- and high-risk colorectal polyps (0.871 and 0.832 respectively).

##### HSUVs and rectal cancer

HSUVs for hypothetical health states related to therapy for locally recurrent rectal cancer were higher among rectal cancer patients than health care professionals when measured using SG [7].

Two sets of rectal cancer-related HSUVs were reported at different time points and at different levels of surgery using EQ-5D and TTO values assigned by the UK general public [66, 67]. However, no standard deviation of mean values was reported. Overall improved survival outweighed the disutility related to AEs of preoperative radiotherapy compared with surgery alone [18, 27]. A summary of rectal cancer-related HSUVs is presented in Table 3.

**Table 3:** Rectal cancer-related HSUVs

Valuation methods used	HSUVs reported	Reference
SG	Healthcare professionals; patients Disease recurrence 0.69; 0.72 Surgical resection 0.69; 0.83 Pain and complications 0.50; 0.78	Miller [7]
EQ-5D	PRT + TME 0.70-0.86 Recurrent 0.67 (local) 0.70 (distant) 0.48 (local/distant) TME 0.63-1.0 Recurrent 0.80 (local) 0.64 (distant) 0.45 (local/distant)	Van den Brink [27]^a^
EQ-5D	Mean EQ-5D (SD) Baseline before TME 0.88 (0.15) 6 weeks after TME 0.85(0.18) 12 weeks after TME 0.87 (0.19) 26 weeks after TME 0.88 (0.17) 52 weeks after TME 0.86 (0.6)	Hompes [18]

^a^ Ranges of reported HSUVs

*HSUVs* health state utility values, *SD* standard deviation, *SG* standard gamble, *PRT* preoperative radiotherapy, *TME* total mesorectal excision

##### HSUVs and AEs/treatments of CRC

Best et al. [4] elicited preferences for seven health states associated with stage III colon cancer and adjuvant chemotherapy using TTO among CRC patients and community members. The TTO values for mCRC obtained from CRC patients were higher than those obtained from the community members. Several CRC health states were measured among CRC patients in Finland and were valued using the UK TTO tariff [51].

Skin toxicity is a common AE related to epidermal growth factor receptor (EGFR) agents. Improved HSUVs related to an EGFR were demonstrated using HUI3 among mCRC patients when compared with best supportive care. Health-related quality of life (HRQL) was measured in mCRC patients and valued by the public [37]. Skin toxicity associated with mCRC treatments was reported to have little impact on HRQoL among mCRC patients [56, 58]. HSUVs obtained from patients with or without anti-EGFR treatment were applied to the duration of the AEs (days with grade 3 or higher AEs) and time without symptoms or toxicity (TWiST), and the differences were measured using a quality-adjusted time without symptoms of disease or toxicity of treatment (Q-TWiST) analysis [60]. Q-TWiST analysis was used to estimate utility values for three health states among CRC patients with liver metastasis undergoing hepatic resection [29].

HRQoL measured directly from patients is not always possible in mCRC; so around 30 carers were used a number of times as a proxy because terminally ill mCRC patients would have difficulties in understanding SG or TTO techniques [20, 36, 48–50]. A summary of HSUVs associated with CRC treatments and AEs are presented in Table 4.

**Table 4:** HSUVs associated with CRC treatments and AEs

Reference	Valuation methods used	HSUVs
Bennett [56]	EQ-5D	1st line Panitimumab + FOLFOX4 0.778; FOLFOX4 0.756 2nd line Panitimumab + FOLFIRI 0.769; FOLFIRI 0.762
Best [4]	TTO	CRC patients/community members Remission 0.83/0.82 adjuvant, no neuropathy 0.61/0.60 adjuvant, mild neuropathy 0.61/0.51 adjuvant, moderate neuropathy 0.53/0.46 adjuvant, severe neuropathy 0.48/0.34 metastatic, stable 0.40)/0.51 metastatic, progressive 0.37/0.21
Dranitsaris [48]^b^	TTO	FOLFOX + ‘new drug’ → FOLFIRI → BSC until death [2–33 months] 0.68-0.89 FOLFOX → FOLFIRI → BSC until death [2–32 months] 0.70-0.94
Dranitsaris [36]^b^	TTO	FOLFOX±’new drug’ → FOLFIRI → BSC until death [2–29 months] 0.67-0.83 FOLFOX → FOLFIRI → BSC until death [2–32 months] 0.72-0.91
Dranitsaris [48]^b^	TTO	FOLFOX + ‘new drug’ → FOLFIRI → BSC until death [2–29 months] 0.52-0.84 FOLFOX → FOLFIRI → BSC until death [2–32 months] 0.53-0.84
Dranitsaris [36]^b^	TTO	FOLFOX + ‘new drug’ → FOLFIRI → BSC until death [2–28 months] 0.44-0.72 FOLFOX → FOLFIRI → BSC until death [2–32 months] 0.44-0.71
Farkkila [51]	EQ-5D	Metastatic disease 0.820 Palliative care 0.643
Mittmann [38]^b^	HUI3	Cetuximab + BSC 0.71-0.77 BSC 0.66-0.71
Odom [58]	EQ-5D	Panitumumab plus BSC; BSC alone Overall 0.72; 0.68 Wild-type KRAS 0.73; 0.68 Mutant KRAS 0.71; 0.68
Petrou [20]^a^	SG	Partial response 1.0 Stable disease 0.95 Progressive disease 0.575 Terminal disease 0.1
Shiroiwa [44]	TTO	XELOX without AEs 0.59 FOLFOX without AEs 0.53 Febrile neutropenia 0.39 Nausea/vomiting 0.38 Diarrhoea 0.42 Hand-foot syndrome 0.39 Fatigue 0.45 Peripheral neuropathy 0.45 Stomatitis 0.42
Wang [60]	EQ-5D	Panitumumab + BSC; BSC TOX 0.6008; 0.4409 TWiST 0.7678; 0.6630 REL 0.6318; 0.6407
Wiering [29]	EQ-5D	Disease-free 0.78 non-curative 0.67 recurrence 0.74 recurrence with chemotherapy 0.82 Recurrent without chemotherapy 0.68
Ward [14]	EQ-VAS	Capecitabine and bevacizumab Baseline 61.76 (SD 23.15) Cycle 2 68.59 (SD 22.26) [p = 0.06] End of study 66.54 (SD 23.18) [p = 0.29]

^a^Reported HSUVs are re-expressed on a 0–1 scale

^b^Ranges of reported HSUVs

*AE* adverse event, *BSC* best supportive care, *FOLFOX* Oxaliplatin + infusional 5 fluorouracil (5-FU), *FOLFIRI* Irinotecan + infusional 5 fluorouracil (5-FU), *FOLFOX4* 5-fluorouracil/folic acid and oxaliplatin, *HSUVs* health state utility values, *KRAS* Kirsten rat sarcoma viral oncogene, *REL* (relapse period until death or end of follow-up), *SD* standard deviation, *SG* Standard gamble, *TOX* days with ≥ grade 3 adverse events, *TTO* time trade-off, *TWiST* time without symptoms or toxicity, *XELOX* capecitabine plus oxaliplatin

Ness et al. [8] reported much lower HSUVs for mCRC than did other studies [10, 11]. People who previously underwent the removal of colorectal adenomas assigned a much lower value to mCRC of 0.25 [8] compared to CRC survivors 0.81 [10] and 0.85 [11]. CRC patients assigned relatively higher values to mCRC (0.820) and palliative care (0.643) compared to those who had no history of previous or current CRC [51]. Stable and progressive disease states were given a much higher value using SG by people who had colorectal adenomas removed [8] compared with those with CRC using TTO [4].

Of five studies reporting HSUVs of different CRC stages HSUVs were clustered ranging from 0.732–0.87 with an exception of one study 0.25–0.74 [8, 10, 11, 22, 33]. A summary of selected HSUVs in different CRC stages is presented in Table 5.

**Table 5:** HSUVs in CRC

Valuation methods used	Reported HSUVs	Reference
SG	Stage I 0.74 Stage II 0.74 (0.59^a^) Stage III 0.67 (0.59^a^) Stage IV 0.25	Ness [8]
HUI3	Stage I 0.84 Stage II 0.86 Stage III 0.85 Stage IV 0.84	Ramsey [10]
HUI3	Stage I 0.83 Stage II 0.86 Stage III 0.87 Stage IV 0.81	Ramsey [10]
EQ-5D	Dukes stage A + B 0.786^b^ Dukes stage C + D 0.806^b^	Wilson [22]^a^
Mapping from FACT-C to SF-6D	Stage I 0.831 Stage II 0.858 Stage III 0.817 Stage IV 0.732	Wong [30]

^a^Rectal cancer; SG Standard gamble

^b^Re-expressed on a 0–1 scale

*FACT*-*C* functional assessment of cancer therapy-cancer

### Discussion

There is no shortage of HSUVs available for those wishing to estimate the cost-effectiveness of diagnostic and treatment strategies with respect to CRC. Those assessing cost-effectiveness face a number of challenges: first, justifying their selection of values when there is no set of values that are clearly superior to all others, and second, negotiating trade-offs between the advantages and disadvantages of the available values.

This choice can be simplified where there is an agreed hierarchy regarding the appropriateness of different approaches to generating HSUVs. In order to aid resource allocation and decision making within a tax-funded healthcare system, economic evaluation needs population values for specific health states related to CRC. The preferences of the public are generally deemed appropriate when health services are largely paid for by taxpayers [59]. Some agencies when have a preference for generic PBMs being used to report the experience of patients in the trial from which the effectiveness of the treatment is being estimated when deciding whether or not to recommend a new treatment, or in the absence of such data similar measures reported in the literature would be used [68].

Researchers usually confront a series of trade-offs and must make judgements about the importance of having all HSUVs used in an economic evaluation come from the same source, or at least obtaining all the HSUVs using the same methods. The number of HSUVs required will in part depend on where in the clinical pathway the intervention being assessed is located. The earlier in the pathway, the larger the number of potentially relevant health states and the less likely it is that all the required HSUVs can come from a single study. Even with clear preferences over the type of measure and the source of values the decision over which values to use can be challenging since the ranking of methods or sources might change in particular circumstances. For example, trial data is not always to be preferred to observational data if the latter provide much larger numbers of observations and are more representative of patients in routine clinical practice. Also a directly collected generic PBM might not always be preferred to the same measure obtained through mapping, for example, if the latter allowed the valuation of a wider range of CRC-related health states. It is uncertain if HSUVs related to mCRC valued by CRC survivors are more relevant than those by health care professionals when making decisions. Also whether or not HSUVs for mCRC valued by early CRC patients are more reliable than those valued by patients with different types of metastatic cancer, has received little attention.

HSUVs have been measured by a surrogate group such as oncology nurses, pharmacists or other health care professionals [20, 36, 48–50]. Despite limitations to the study design (such as small sample size or under-explored uncertainties) these HSUVs continue to be used in economic evaluation studies associated with CRC [20]. Subsequently, these uncertainties are inherited by the estimation of cost-effectiveness and of QALYs. Well-designed clinical studies continue to generate new evidence that is highly focussed on treatment effects with strong internal validity. Economic evaluation would be strengthened if health state data could be taken from the clinical studies that provide the estimates of effectiveness of treatment [69]. Further research which utilises data from patient-reported outcomes, population surveys, and cancer registry data in assessing HRQoL and HSUVs is recommended [70].

Given the absence of generic PBM data from many trials, existing mapping algorithms could be more fully utilised as an additional means of deriving HSUVs for economic evaluation of CRC, and also exploratory studies to derive HSUVs for colorectal cancer health states from EORTC QLQ-C30 data using the EORTC-8D or QLU-C10D are warranted.

Although there are a number of algorithms for mapping from cancer-specific scales to generic PBMs this approach has not been frequently reported with respect to CRC. Cancer-specific scales, such as the QLQ-C30, capture a number of clinical and domain-specific effects that might not be captured when using generic PBMs [68]. Mapping also has the advantage of producing QALYs measured using a familiar metric. However, any mapping inevitably introduces additional uncertainty to the QALY calculation and the cost-effectiveness estimation.

Important questions associated with HSUVs for the economic evaluation of CRC remain unanswered. What is the most accurate way of measuring and valuing HRQoL in CRC? Is it better to collect HRQoL data directly from a small number of CRC patients over a follow-up period [27]? The studies reviewed gave limited consideration to the best way to measure and value CRC health states.

This review highlights gaps in the evidence and opportunities for informative research. The most appropriate way to measure and value CRC-related health states should be studied. Developing a set of criteria for selecting the most appropriate HSUVs that fits the analyst’s purpose is encouraged. It is not known whether the mCRC-related HRQoL of CRC survivors is more representative than those derived from a small surrogate group. Also, whether mCRC-related HSUVs valued by early CRC patients are more appropriate than those valued by patients with different types of metastatic cancer for economic evaluation has been under-researched.

## Conclusions

CRC-related HSUVs vary markedly between studies and across methods. Despite the number of HSUVs published, there is not a set of HSUVs that are methodologically robust with a full range of values for health states of interest appropriate for the use in economic evaluation of CRC. There is considerable scope for new HSUVs to be developed which improve on those currently available and consequently to produce better estimates of QALYs and cost-effectiveness in order to better inform resource allocation and healthcare decision making. In addition, the use of existing mapping algorithms to derive CRC-related HSUVs should be further explored.

## Appendix 1. Search strategies

### Appendix 1.1 Search strategy for MEDLINE

Database: Ovid MEDLINE(R), Ovid MEDLINE(R) In-Process & Other Non-Indexed Citations, Ovid MEDLINE(R) Daily and Ovid OLDMEDLINE(R) <1946 to Present>

Platform used: OvidSP.

Date run: 30 October 2015

Search Strategy:

1. Economics/or exp "Costs and Cost Analysis"/or exp Economics, Hospital/or exp Economics, Medical/or Economics, Nursing/or Economics, Pharmaceutical/or Budgets/or exp Models, Economic/or Markov Chains/or Monte Carlo Method/or Decision Trees/
2. (econom$ or cba or cea or cua or markov$ or (monte adj carlo) or (decision adj2 (tree$ or analys$)) or (cost or costs or costing$ or costly or costed) or (price$ or pricing$) or budget$ or expenditure$ or (value adj2 (money or monetary)) or (pharmacoeconomic$ or (pharmaco adj economic$))).ti,ab.
3. 1 or 2
4. "Value of Life"/
5. Quality-Adjusted Life Years/
6. quality adjusted life year.tw.
7. (qaly$ or qald$ or qale$ or qtime$).tw.
8. disability adjusted life.tw.
9. daly$.tw.
10. Health Status Indicators/
11. (sf36 or sf 36 or short form 36 or shortform 36 or sf thirtysix or sf thirty six or shortform thirtysix or shortform thirty six or short form thirtysix or short form thirty six).tw.
12. (sf6 or sf 6 or short form 6 or shortform 6 or sf six or sfsix or shortform six or short form six).tw.
13. (sf12 or sf 12 or short form 12 or shortform 12 or sf twelve or sftwelve or shortform twelve or short form twelve).tw.
14. (sf16 or sf 16 or short form 16 or shortform 16 or sf sixteen or sfsixteen or shortform sixteen or short form sixteen).tw.
15. (sf20 or sf 20 or short form 20 or shortform 20 or sf twenty or sftwenty or shortform twenty or short form twenty).tw.
16. (euroqol or euro qol or eq5d or eq 5d).tw.
17. (qol or hql or hqol or hrqol).tw.
18. (hye or hyes).tw.
19. health$ year$ equivalent$.tw.
20. utilit$.tw.
21. (hui or hui$1 or hui$2 or hui$3).tw.
22. disutili$.tw.
23. rosser.tw.
24. quality of wellbeing.tw.
25. quality of well-being.tw.
26. qwb.tw.
27. willingness to pay.tw.
28. standard gamble$.tw.
29. time trade off.tw.
30. time tradeoff.tw.
31. tto.tw.
32. mapping.tw.
33. mapped.tw.
34. crosswalk.tw.
35. transfer$ to utilit$.tw.
36. or/4–35
37. ((colorectal or colon$ or rectal or rectum$) and (cancer$ or tumor$ or tumor$ or neoplasm$ or carcinoma$ or adenoma$)).tw.
38. Colorectal Neoplasms/or Colonic Neoplasms/or rectal neoplasms/
39. crc.tw.
40. 37 or 38 or 39
41. 36 and 40
42. 3 and 41
43. limit 42 to humans

### Appendix 1.2 Search strategy for mapping studies - MEDLINE

Database: Ovid MEDLINE(R) In-Process & Other Non-Indexed Citations, Ovid MEDLINE(R) Daily, Ovid MEDLINE(R) and Ovid OLDMEDLINE(R) <1946 to Present>

Platform used: OvidSP. Date run: 30 October 2015

Search Strategy:

1. ((colorectal or colon$ or rectal or rectum$) and (cancer$ or tumor$ or tumor$ or neoplasm$ or carcinoma$ or adenoma$)).tw.
2. Colorectal Neoplasms/or Colonic Neoplasms/or rectal neoplasms/
3. crc.tw.
4. or/1–3
5. mapping$.tw.
6. mapped$.tw.
7. (crosswalk$ or cross walk$).tw.
8. transfer$ to utilit$.tw.
9. (euroqol or euro qol or eq5d or eq 5d).tw.
10. (sf36 or sf 36 or short form 36 or shortform 36 or sf thirtysix or sf36 or shortform thirtysix or shortform 36 or short form thirtysix or short form 36).tw.
11. (sf6 or sf 6 or short form 6 or shortform 6 or sf six or sfsix or shortform six or short form six).tw.
12. (sf12 or sf 12 or short form 12 or shortform 12 or sf 12 or sftwelve or shortform 12 or short form 12).tw.
13. (sf16 or sf 16 or short form 16 or shortform 16 or sf 16 or sfsixteen or shortform 16 or short form 16).tw.
14. (sf20 or sf 20 or short form 20 or shortform 20 or sf 20 or sftwenty or shortform 20 or short form 20).tw.
15. or/5–14
16. 4 and 15
17. limit 16 to humans
18. remove duplicates from 17

## Appendix 2. Study selection criteria

Studies were excluded if;

- the title/abstract were irrelevant to HRQoL or CRC-related HSUVs
- conference abstracts with no full publication
- Psychometric validation studies or description of health states without interval properties rather than valuation of health states
- values were previously reported in other included studies
- unspecified/not clearly specified health states relating to CRC
- primary mapping function is not reported in studies

Studies were included if;

- CRC-related HSUVs which were had not been reported previously which were
  - ○ preference-based generic measure such as EQ-5D, HUI3, and SF-6D
  - ○ or directly valued health state descriptions
  - ○ or mapping to generic preference-based measures based on direct statistical association mapping

## Additional files

Additional file 1: Included studies in the review. (DOCX 17.5 KB) Additional file 2: Summary of reporting HSUVs in CRC. (XLSX 17.6 KB) Additional file 3: Summary of mapping studies. (XLSX 9.22 KB)

## Abbreviations

AE: Adverse event
CRC: Colorectal cancer
EGFR: Epidermal growth factor receptor
EORTC: European Organisation for Research and Treatment of Cancer
FACT-G: Functional assessment of cancer therapy-general
FACT-C: Functional assessment of cancer therapy-cancer
HRQoL: Health-related quality of life
HSUV: Health state utility value
mCRC: Metastatic colorectal cancer
PBM: Preference-based measure
QLQ: Quality of life questionnaire
QALY: Quality-adjusted life-year
Q-TWiST: Quality-adjusted time without symptoms or toxicity
NICE: National Institute for Health and Care Excellence
SG: Standard gamble
TTO: Time trade-off
TWiST: Time without symptoms or toxicity
UK: United Kingdom
US: United States
