# Supplementary material for: Systematic review of health state utility values for economic evaluation of colorectal cancer
Source: Health Econ Rev. 2016 Aug 19;6:36. doi: 10.1186/s13561-016-0115-5 (PMC4991979; doi:10.1186/s13561-016-0115-5)
Supplement: Additional file 1: — Included studies in the review. (DOCX 17.5 KB) [file 13561_2016_115_MOESM1_ESM.docx]

Appendix 3. Studies included in the review

1. Boyd, N.F., et al., *Whose utilities for decision analysis?* Medical Decision Making, 1990. **10**(1): p. 58-67.

2. Smith, R., et al., *A cost-utility approach to the use of 5-fluorouracil and levamisole as adjuvant chemotherapy for Dukes' C colonic carcinoma.* Medical Journal of Australia, 1993. **158:319-322**.

3. Dominitz, J.A. and D. Provenzale, *Patient preferences and quality of life associated with colorectal cancer screening.* Am J Gastroenterol, 1997. **92**(12): p. 2171-8.

4. Norum, J., et al., *Adjuvant chemotherapy (5-fluorouracil and levamisole) in Dukes' B and C colorectal carcinoma. A cost-effectiveness analysis.* Annals of Oncology, 1997. **8**(1): p. 65-70.

5. Petrou, S. and N. Campbell, *Stabilisation in colorectal cancer.* International Journal of Palliative Nursing, 1997. **3**(5): p. 275.

6. Syngal, S., et al., *Benefits of colonoscopic surveillance and prophylactic colectomy in patients with hereditary nonpolyposis colorectal cancer mutations.* Annals of Internal Medicine, 1998. **129**(10): p. 787-96.

7. Ness, R.M., et al., *Utility valuations for outcome states of colorectal cancer.* American Journal of Gastroenterology, 1999. **94**(6): p. 1650-7.

8. Miller, A., et al., *Quality of life and cost effectiveness analysis of therapy for locally recurrent rectal cancer.* Diseases of the Colon and Rectum, 2000. **1695-1703**.

9. Ramsey, S.D., et al., *Quality of life in survivors of colorectal carcinoma.* Cancer, 2000. **88**(6): p. 1294-1303.

10. Hamashima, C., *Long-term quality of life of postoperative rectal cancer patients.* Journal of Gastroenterology & Hepatology, 2002. **17**(5): p. 571-6.

11. Ramsey, S.D., et al., *Quality of life in long term survivors of colorectal cancer.* Am J Gastroenterol, 2002. **97**(5): p. 1228-34.

12. van den Brink, M., et al., *Cost-utility analysis of preoperative radiotherapy in patients with rectal cancer undergoing total mesorectal excision: a study of the Dutch Colorectal Cancer Group.* Journal of clinical oncology : official journal of the American Society of Clinical Oncology, 2004. **22**(2): p. 244-253.

13. Gosselink, M.P., et al., *Quality of life after total mesorectal excision for rectal cancer.* Colorectal Disease, 2006. **8**(1): p. 15-22.

14. Smith, D.M., et al., *Misremembering colostomies? Former patients give lower utility ratings than do current patients.* Health Psychol, 2006. **25**(6): p. 688-95.

15. Wilson, T.R., D.J. Alexander, and P. Kind, *Measurement of health-related quality of life in the early follow-up of colon and rectal cancer.* Diseases of the Colon & Rectum, 2006. **49**(11): p. 1692-702.

16. Doornebosch, P.G., et al., *Quality of life after transanal endoscopic microsurgery and total mesorectal excision in early rectal cancer.* Colorectal Disease, 2007. **9**(6): p. 553-558.

17. Sharma, A., et al., *Predictors of early postoperative quality of life after elective resection for colorectal cancer.* Annals of Surgical Oncology, 2007. **14**(12): p. 3435-3442.

18. Doornebosch, P.G., et al., *Impact of transanal endoscopic microsurgery on functional outcome and quality of life.* International Journal of Colorectal Disease, 2008. **23**(7): p. 709-713.

19. Cheung, Y.B., et al., *Mapping the English and Chinese versions of the Functional Assessment of Cancer Therapy-General to the EQ-5D utility index.* Value Health, 2009. **12**(2): p. 371-6.

20. Mittmann, N., et al., *Prospective cost-effectiveness analysis of cetuximab in metastatic colorectal cancer: Evaluation of national cancer institute of canada clinical trials group CO.17 Trial.* Journal of the National Cancer Institute, 2009. **101**(17): p. 1182-1192.

21. Shiroiwa, T., T. Fukuda, and K. Tsutani, *Cost-effectiveness analysis of XELOX for metastatic colorectal cancer based on the NO16966 and NO16967 trials.* British Journal of Cancer, 2009. **101**(1): p. 12-8.

22. Best, J.H., et al., *Preference values associated with stage III colon cancer and adjuvant chemotherapy.* Quality of Life Research, 2010. **19**(3): p. 391-400.

23. Wiering, B., et al., *Added value of positron emission tomography imaging in the surgical treatment of colorectal liver metastases.* Nuclear Medicine Communications, 2010. **31**(11): p. 938-44.

24. Bennett, L., et al., *Health-related quality of life in patients with metastatic colorectal cancer treated with panitumumab in first- or second-line treatment.* Br J Cancer, 2011. **105**(10): p. 1495-502.

25. Haapamaki, M.M., et al., *Physical performance and quality of life after extended abdominoperineal excision of rectum and reconstruction of the pelvic floor with gluteus maximus flap.* Diseases of the Colon & Rectum, 2011. **54**(1): p. 101-6.

26. Hornbrook, M.C., et al., *Complications among colorectal cancer survivors: SF-6D preference-weighted quality of life scores.* Medical Care, 2011. **49**(3): p. 321-326.

27. Odom, D., et al., *Health-related quality of life and colorectal cancer-specific symptoms in patients with chemotherapy-refractory metastatic disease treated with panitumumab.* Int J Colorectal Dis, 2011. **26**(2): p. 173-81.

28. Wang, J., et al., *A Q-TWiST analysis comparing panitumumab plus best supportive care (BSC) with BSC alone in patients with wild-type KRAS metastatic colorectal cancer.* Br J Cancer, 2011. **104**(12): p. 1848-53.

29. Wiering, B., et al., *Long-term global quality of life in patients treated for colorectal liver metastases.* British Journal of Surgery, 2011. **98**(4): p. 565-71; discussion 571-2.

30. Dranitsaris, G., et al., *Improving patient access to cancer drugs in India: Using economic modeling to estimate a more affordable drug cost based on measures of societal value.* International Journal of Technology Assessment in Health Care, 2011a. **27**(1): p. 23-30.

31. Dranitsaris, G., et al., *Using pharmacoeconomic modelling to determine value-based pricing for new pharmaceuticals in Malaysia.* Malaysian Journal of Medical Sciences, 2011b. **18(4):31-42**.

32. Kapidzic, A., et al., *Quality of life in participants of a CRC screening program.* Br J Cancer, 2012. **107**(8): p. 1295-301.

33. Kim, S.H., et al., *Mapping EORTC QLQ-C30 onto EQ-5D for the assessment of cancer patients.* Health Qual Life Outcomes, 2012. **10**: p. 151.

34. Pickard, A.S., et al., *Comparison of FACT- and EQ-5D-based utility scores in cancer.* Value Health, 2012. **15**(2): p. 305-11.

35. Wong, C.K., et al., *Mapping the Functional Assessment of Cancer Therapy-general or -Colorectal to SF-6D in Chinese patients with colorectal neoplasm.* Value in Health, 2012. **15**(3): p. 495-503.

36. Dranitsaris, G., et al., *A pharmacoeconomic modeling approach to estimate a value-based price for new oncology drugs in Europe.* Journal of Oncology Pharmacy Practice, 2012a. **18**(1): p. 57-67.

37. Dranitsaris, G., et al., *The application of pharmacoeconomic modelling to estimate a value-based price for new cancer drugs.* Journal of Evaluation in Clinical Practice, 2012b. **18**(2): p. 343-51.

38. Andersson, J., et al., *Health-related quality of life after laparoscopic and open surgery for rectal cancer in a randomized trial.* British Journal of Surgery, 2013. **100**(7): p. 941-9.

39. Augestad, K., et al., *Cost-effectiveness and quality of life in surgeon versus general practitioner-organised colon cancer surveillance: a randomised controlled trial.* BMJ Open, 2013. **3(4):e002391**.

40. Farkkila, N., et al., *Health-related quality of life in colorectal cancer.* Colorectal Disease, 2013. **15**(5): p. e215-e222.

41. Lee, L., et al., *Valuing postoperative recovery: validation of the SF-6D health-state utility.* Journal of Surgical Research, 2013. **184**(1): p. 108-114.

42. Schwandner, O., *Sacral neuromodulation for fecal incontinence and "low anterior resection syndrome" following neoadjuvant therapy for rectal cancer.* International Journal of Colorectal Disease, 2013. **28**(5): p. 665-9.

43. Wong, C.K., et al., *Predicting SF-6D from the European Organization for Treatment and Research of Cancer Quality of Life Questionnaire scores in patients with colorectal cancer.* Value in Health, 2013a. **16**(2): p. 373-84.

44. Wong, C.K., et al., *Clinical correlates of health preference and generic health-related quality of life in patients with colorectal neoplasms.* PLoS ONE [Electronic Resource], 2013b. **8**(3): p. e58341.

45. Brown, S.R., et al., *The impact of postoperative complications on long-term quality of life after curative colorectal cancer surgery.* Annals of Surgery, 2014. **259**(5): p. 916-23.

46. Carter, H.E., et al., *The cost effectiveness of bevacizumab when added to capecitabine, with or without mitomycin-C, in first line treatment of metastatic colorectal cancer: Results from the Australasian phase III MAX study.* European journal of cancer (Oxford, England : 1990), 2014. **50**(3): p. 535-543.

47. Jordan, J., et al., *Laparoscopic versus open colorectal resection for cancer and polyps: A cost-effectiveness study.* ClinicoEconomics and Outcomes Research, 2014. **6**: p. 415-422.

48. Kim, S.H., et al., *Deriving a mapping algorithm for converting SF-36 scores to EQ-5D utility score in a Korean population.* Health and quality of life outcomes, 2014. **12**: p. 145.

49. Polat, U., et al., *Evaluation of quality of life and anxiety and depression levels in patients receiving chemotherapy for colorectal cancer: Impact of patient education before treatment initiation.* Journal of Gastrointestinal Oncology, 2014. **5**(4): p. 270-275.

50. Stein, D., et al., *Assessing health-state utility values in patients with metastatic colorectal cancer: a utility study in the United Kingdom and the Netherlands.* International Journal of Colorectal Disease, 2014. **29**(10): p. 1203-10.

51. Ward, P., et al., *Physical function and quality of life in frail and/or elderly patients with metastatic colorectal cancer treated with capecitabine and bevacizumab: an exploratory analysis.* Journal of Geriatric Oncology, 2014. **5**(4): p. 368-75.

52. Wong, C.K., et al., *Responsiveness was similar between direct and mapped SF-6D in colorectal cancer patients who declined.* Journal of Clinical Epidemiology, 2014. **67**(2): p. 219-27.

53. Yang, Y., et al., *Improving the mapping of condition-specific health-related quality of life onto SF-6D score.* Quality of life research : an international journal of quality of life aspects of treatment, care and rehabilitation, 2014. **23**(8): p. 2343-2353.

54. Downing, A., et al., *Health-related quality of life after colorectal cancer in England: a patient-reported outcomes study of individuals 12 to 36 months after diagnosis.* Journal of Clinical Oncology, 2015. **33**(6): p. 616-24.

55. Hall, P.S., et al., *Costs of cancer care for use in economic evaluation: a UK analysis of patient-level routine health system data.* British Journal of Cancer, 2015. **112**(5): p. 948-56.

56. Hompes, R., et al., *Evaluation of quality of life and function at 1 year after transanal endoscopic microsurgery.* Colorectal Disease, 2015. **17**(2): p. O54-O61.

57. Young, C.J., et al., *Improving Quality of Life for People with Incurable Large-Bowel Obstruction: Randomized Control Trial of Colonic Stent Insertion.* Diseases of the Colon & Rectum, 2015. **58**(9): p. 838-49.
